# Improved estimation of the relationship between fetal growth and late stillbirth in the United States, 2014–15

**DOI:** 10.1038/s41598-024-56572-7

**Published:** 2024-03-12

**Authors:** Darren Tanner, Juan M. Lavista Ferres, Edwin A. Mitchell

**Affiliations:** 1 AI for Health, AI for Good Research Lab, Microsoft Corporation, Redmond, WA USA; 2 Department of Paediatrics: Child and Youth Health, The University of Auckland, Auckland, New Zealand

**Keywords:** Epidemiology, Risk factors

## Abstract

In the United States the rate of stillbirth after 28 weeks’ gestation (late stillbirth) is 2.7/1000 births. Fetuses that are small for gestational age (SGA) or large for gestational age (LGA) are at increased risk of stillbirth. SGA and LGA are often categorized as growth or birthweight ≤ 10th and ≥ 90th centile, respectively; however, these cut-offs are arbitrary. We sought to characterize the relationship between birthweight and stillbirth risk in greater detail. Data on singleton births between 28- and 44-weeks’ gestation from 2014 to 2015 were extracted from the US Centers for Disease Control and Prevention live birth and fetal death files. Growth was assessed using customized birthweight centiles (Gestation Related Optimal Weight; GROW). The analyses included logistic regression using SGA/LGA categories and a generalized additive model (GAM) using birthweight centile as a continuous exposure. Although the SGA and LGA categories identified infants at risk of stillbirth, categorical models provided poor fits to the data within the high-risk bins, and in particular markedly underestimated the risk for the extreme centiles. For example, for fetuses in the lowest GROW centile, the observed rate was 39.8/1000 births compared with a predicted rate of 11.7/1000 from the category-based analysis. In contrast, the model-predicted risk from the GAM tracked closely with the observed risk, with the GAM providing an accurate characterization of stillbirth risk across the entire birthweight continuum. This study provides stillbirth risk estimates for each GROW centile, which clinicians can use in conjunction with other clinical details to guide obstetric management.

## Introduction

The World Health Organization estimates that there are nearly 2 million stillbirths after 28 weeks’ gestation each year (late stillbirth)^1^. In the United States (US), among pregnancies that reach at least 28 weeks’ gestation, the stillbirth rate stands at approximately 2.7 stillbirths/1000 births^2,3^. This is higher than in many other high income countries^4^, suggesting that there are opportunities to reduce stillbirth rates.

One of the strongest documented risk factors for stillbirth is abnormal fetal growth, where infants who are small for gestational age (SGA) are at particularly high risk of being stillborn. Infants who are large for gestational age (LGA) have also been reported to be at higher risk of stillbirth, particularly at later gestational periods, though the magnitude of risk associated LGA is not as large as that for SGA^5,6^. For example, in a meta-analysis of risk factors for stillbirth, including three studies from high-income countries, Flenady et al.^7^ reported a pooled adjusted odds ratio of 3.9 (CI 3.9–5.1) of stillbirth for SGA infants. In previous related work using US Centers for Disease Control and Prevention (CDC) data^3^, our group found adjusted risk ratios of 5.43 (CI 5.27–5.60) and 1.68 (CI 1.61–1.76), where 38% and 6% of stillbirths could be attributed SGA and LGA, respectively. This is consistent with an autopsy study of clinically unexplained stillbirths which found that 37% were due to fetal growth restriction^8^.

Many studies use birthweight to examine risk associated with abnormal fetal growth, where SGA and LGA are defined by risk category bins based on birthweight centile charts. Following Battaglia and Lubchenco^9^, most have used bin cutoffs for SGA and LGA of ≤ 10th and ≥ 90th centile for birthweight, respectively, adjusted for infant sex and gestational age, though customized birthweight standards adjust for further factors, including maternal race, height, weight, and parity^10–15^.

However, the 10th/90th percentile cutoffs for SGA and LGA in assessing perinatal risk, including stillbirth, may be arbitrary^16^. Indeed, some researchers vary with regard to where SGA/LGA cutoffs should be set, or whether LGA infants are at increased risk of perinatal mortality and stillbirth at all^6,16–25^. Moreover, using categorical cutoffs and binning for defining SGA and LGA, as is common practice in the field, does not adequately capture the relationship between adjusted birthweight centiles and stillbirth risk, which is not categorical but continuous and nonlinear.

This study aimed to characterize the relationship between birthweight and risk for stillbirth using both traditional generalized linear models (GLMs) and a generalized additive model (GAM). GAMs provide interpretable models for complex, non-linear relationships between a predictor variable (birthweight centile) and outcome (stillbirth) using smooth functions. GAMs can give a continuous and detailed view of the relationship between birthweight and stillbirth risk without the need for ad-hoc researcher-defined bins.

## Methods

### Data and study population

**Figure 1:**
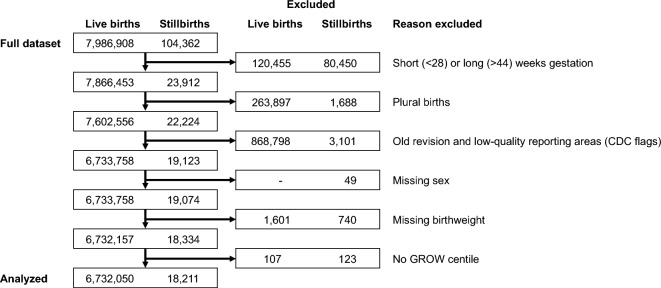
Case flow information. GROW centile reflects customized birthweight centile using Gestation Related Optimal Weight standard.

The data source and case selection method is described in detail in our prior publication^3^. Reporting of this study follows the REporting of studies Conducted using Observational Routinely-collected health Data Statement^26^. In brief, data were extracted from the 2014–2015 US CDC live birth and fetal death files^27,28^ and were subset to all births between 28 and 44 weeks’ gestation, inclusive. We subset to only those observations that used the 2003 revised versions of the US Standard Report of Fetal Death and US Standard Certificate of Live birth as opposed to the unrevised 1989 versions and further excluded observations missing infant birthweight or infant sex, plural births, as well as those from areas flagged by the CDC as having low reporting quality. We additionally excluded observations with birthweights below 150 g or above 8000 g, as no birthweight centile score could be calculated for these birthweight extremes (see below). Case flow information is provided in Fig. 1. We used publicly available data, so IRB approval was not required.

### Exposure variable

The exposure variable of interest in this study was infant birthweight centile, calculated using customized centile scores, as defined by the gestation related optimal weight (GROW) standard^11^. GROW computes centile scores with values between 0 and 100 (inclusive) by adjusting birthweight for gestational age, maternal country of origin/race, maternal height and prepregnancy weight, infant sex, parity, and outcome (live birth or fetal death). In cases involving stillbirth, two days are subtracted from the gestational age at birth to account for delays between fetal death and delivery. For the present work, we used customized as opposed to uncustomized centiles as there is substantial variation in infant birthweight in healthy, uncomplicated pregnancies that co-varies systematically with the adjustment variables, but where this systematic co-variation is not associated with increased pathology^12,14^. Very low and very high centiles after these adjustments are therefore more likely to be indicative of true fetal growth restriction or macrosomia compared with uncustomized centiles, and they therefore more precisely indicate infants at risk^10–15^. For GROW centiles, the GROW calculation software (v8.0.6.1)^29^ used the following imputation methods for missing observations, with the imputation strategy and values implemented within the software itself: no centile was calculated if birthweight was less than 150 g or greater than 8000 g; if maternal height and/or weight were missing, the average for the ethnicity/country of origin was used; if either of these values and ethnicity/country of origin were missing, the global average was used; if parity was missing, the value 1 was used^29^. Overall, 3.83% of observations had at least one variable imputed among maternal height, maternal weight, and parity; distributions and counts for the combination of variables imputed are provided in Supplementary Table S1 online.

### Covariates

Unless otherwise specified, all models included the following factors as adjustment covariates for the relationship between birthweight centile and stillbirth risk; these were selected as potential confounders for the relationship between birthweight and stillbirth, and their association with stillbirth had been confirmed in our prior work using this dataset:^3^ maternal age (categorized < 15 years, 15–19, 20–24, 25–29, 30–34, 35–39, 40–44, 45+); paternal age (< 15, 15–19, 20–24, 25–29, 30–34, 35–39, 40–44, 45–49, 50+, unknown or not stated), self-reported maternal educational attainment (8th grade or less, 9th–12th grade, high school graduate or GED, some college, associate degree, bachelor’s degree, master’s degree, doctorate or doctoral-level professional degree, unknown or not stated); self-reported maternal race (White, Black, American Indian/Alaska Native, Asian, Native Hawaiian and Other Pacific Islander, more than one race, unknown or not stated); self-reported maternal Hispanic ethnic origin (non-Hispanic, Mexican, Puerto Rican, Cuban, Central and South American, other and unknown Hispanic origin, unknown or not stated); maternal place of birth (nativity; born in the US (50 states), born outside the US (incl. territories), unknown or not stated); prepregnancy maternal body mass index (BMI; underweight (< 18.5 kg/m^2^), normal (18.5–24.9), overweight (25.0–29.9), obesity I (30.0–34.9), obesity II (35.0–39.9), obesity III (40.0+), unknown or not stated); self-reported participation in the supplemental nutrition for Women, Infants, and Children program (WIC; no, yes, unknown or not stated); self-reported maternal smoking status (did not smoke in the three months prior to or during pregnancy (non-smoker), smoked before and during pregnancy (continued smoking), smoked before pregnancy but stopped by the first trimester (quit smoking), did not smoke before pregnancy but reported smoking at some point during pregnancy (started smoking), unknown or not stated); diabetes (no diabetes diagnosis, prepregnancy diabetes, gestational diabetes, unknown or not stated); hypertension (no diagnosis of hypertension, prepregnancy hypertension only, gestational hypertension only, eclampsia, unknown or not stated); use of infertility treatment (no, yes, unknown or not stated); number of previous live births (0, 1, 2, 3, 4, 5 6, 7+); timing of start of prenatal care (1st–3rd month, 4th–6th, 7th–final month, no prenatal care, unknown or not stated); sex of fetus (female, male).

### Statistical analysis

We fit a series of logistic regression models in R^30^. The outcome (live birth or stillbirth) was modeled using a binomial distribution with a logit link function. Univariate GLMs estimated crude odds ratios (ORs) and multivariate GLMs included all covariates above to estimate adjusted odds ratios (aORs) for stillbirth. The first model was a logistic GLM focusing on stillbirth risk associated with birthweight by using GROW centiles binned with common thresholds: ≤ 10th centile for SGA and ≥ 90th centile for LGA, with those between the 10th and 90th centiles defined as appropriate for gestational age (AGA) and set as the reference level.

To model the relationship between birthweight centile and stillbirth risk in more detail we fit two additional models: one logistic GLM and one logistic GAM. The GLM used more bins across a broader range of GROW values than those used in the first model. The goal was to provide a more fine-grained set of estimates for risk across a broad range of birthweight centiles relative to a reference range in the center of the birthweight centile continuum while still using a traditional logistic regression approach. The reference range was defined as GROW values between the 40th and 60th centiles. The comparison GROW centiles were 5 centile bins (0–4, 5–9, 10–14, 15–19, 20–24, 25–29, 30–34, 35–39, 40–60 (reference), 61–65, 66–70, 71–75, 76–80, 81–85, 86–90, 91–95, 96–100). Unadjusted and adjusted ORs were estimated for each bin, relative to the reference range. As a qualitative assessment, we adapt a heuristic used by Xu et al.^16^ who suggested that an aOR ≥ 2.0 be considered clinically meaningful.

Finally, we fit a GAM using the *mgcv* package^31,32^. GAMs give a continuous estimate of the contribution of birthweight centile to odds of stillbirth without ad-hoc binning. To fit the GAM, we first rounded GROW centiles to integer values between 0 and 100 to reduce complexity of the spline basis space. The model was defined with a smooth term for GROW centile using thin plate splines and parametric terms for the covariates described above. The upper-limit of the basis space (100) was passed to the model as the ‘k’ parameter for the smooth term.

Fits for all models were assessed using area under the receiver operating characteristic curve (AUC), sensitivity, specificity, and positive predictive value (PPV). AUC provides an estimation of the ability of the model to discriminate between stillborn and live born observations; an AUC of 1.0 reflects perfect discrimination and an AUC of 0.5 reflects random chance discrimination. For sensitivity, specificity, and PPV, probabilities > 0.5 from the fitted models were considered to reflect a positive prediction of stillbirth. 95% confidence intervals (CIs) for the AUC, sensitivity, specificity, and PPV metrics were obtained through the stratified bootstrap method; 500 bootstrap iterations were computed for each model to form the empirical distribution for each metric, and CIs were derived by taking the 0.025 and 0.975 quantiles across iterations.

## Results

**Figure 2:**
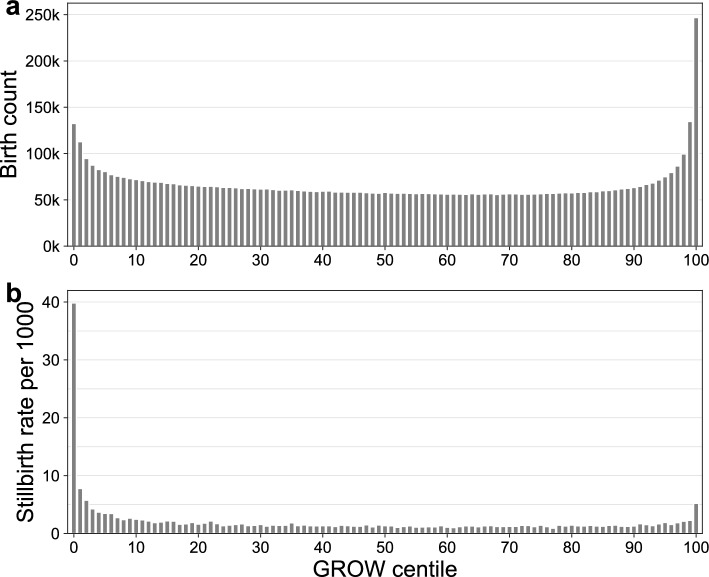
(**a**) Distribution of infant GROW centiles. (**b**) Stillbirth rate per 1000 births by GROW centile.

The overall stillbirth rate in our dataset was 2.7/1000 births. The characteristics of the cohort of births by level of GROW, defined using the ≤ 10th and ≥ 90th cutoffs for the SGA and LGA bins, is provided in Supplementary Table S2 online. The distribution of infants’ GROW centile scores is depicted in Fig. 2a. GROW centiles showed a bimodal distribution, with marked increases in the number of infants at the two ends of the continuum compared to the center of the GROW range. The stillbirth rate per 1000 births as a function of GROW centile is depicted in Fig. 2b. Compared to infants with customized birthweight centiles in the middle of the range, infants with lower birthweight centiles begin showing small risk increases beginning approximately between the 30th and 39th centiles, with a slowly increasing risk function associated with decreasing birthweight. The increase in risk then accelerates beginning below the 15th centile, with a large spike in stillbirth rates in the 0th centile (39.8/1000 births). Conversely, infants with high adjusted birthweights were at modestly increased risk of stillbirth, but this risk increase is only apparent in the very highest GROW centile bin. The stillbirth rate in the 100^th^ centile bin was 5.2/1000 births.

Results from the first logistic GLM using cutoffs of ≤ 10th and ≥ 90th centile for SGA and LGA, respectively are shown in Table 1. In both the unadjusted and adjusted analysis, SGA and LGA infants were at higher odds of stillbirth, with the SGA infants markedly so.

**Table 1:** Number of live births and late stillbirths, late stillbirth rate per 1000 births, and the unadjusted and adjusted^a^ odds ratios (aORs) for GROW birthweight centile categories.

GROW centile bin	Live birth N	Stillbirth N	Stillbirth rate per 1000	OR (95% CI)	aOR (95% CI)
SGA^b^ (≤ 10th centile)	918,394	8,558	9.23	**6.47 (6.27, 6.68)**	**5.72 (5.53, 5.91)**
AGA^c^ (> 10th– < 90th)	4,785,410	6,893	1.44	Ref	Ref
LGA^d^ (≥ 90th centile)	1,028,246	2,760	2.68	**1.86 (1.78, 1.95)**	**1.69 (1.61, 1.77)**

Numbers in bold indicate category is significantly different from the reference category (ref).

^a^aORs in this and all subsequent tables are adjusted for mother’s race, mother’s Hispanic origin, mother’s age, mother’s prepregnancy body mass index (BMI), mother’s educational attainment, mother’s nativity, father’s age, number of previous live births, infertility treatment, diabetes, hypertension, smoking, timing of prenatal care onset, infant sex, and participation in the supplemental nutrition for women infants and children program (WIC).

^b^Small for gestational age.

^c^Appropriate for gestational age.

^d^Large for gestational age.

**Figure 3:**
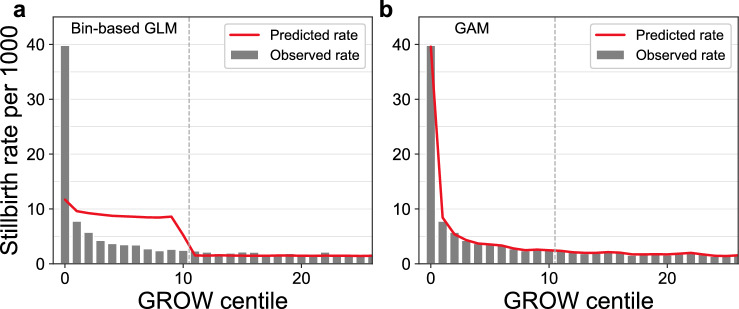
Observed and mean predicted stillbirth rates as a function of GROW centile. (**a**) Observed rates (grey bars) and mean predicted rates (red line) derived from the bin-based logistic GLM using the 10th and 90th centile cutoffs to define SGA and LGA bins, respectively. (**b**) Observed rates (grey bars) and mean predicted rates (red line) derived from GAM. Vertical dashed lines depict the 10th centile SGA bin cutoff used in the logistic GLM. X-axes are limited to GROW values ≤ 25. Plots of full GROW distribution are provided in Supplementary Fig. S1 online.

Although this model captured the increased risk associated with very small and very large infants, as can be seen in Fig. 2b, there is no clear change in observed stillbirth rates at the 10th or 90th centile coinciding with the SGA and LGA category cutoffs. The SGA and LGA bins defined with these cutoffs do not capture the slow, steady increase in stillbirth rates below the 35th centile, nor the sharp increases at the two extreme ends of the GROW continuum. Figure 3a depicts the observed stillbirth rates and predicted rates from this first GLM, with a focus on the low end of the GROW centile distribution where the discrepancy between predicted and observed rates is largest. The sharp change in predicted rate associated with the bin cutoff does not track well with changes in the observed rate, where there is no abrupt increase at the bin edge. This is particularly apparent with the SGA bin depicted in Fig. 3a, where the model over-predicts stillbirth rates between the 1–9th centile and greatly under-predicts rates in the 0^th^ centile. Plots showing observed/predicted rates for the full GROW continuum are provided in Supplementary Fig. S1 online.

In the second GLM, we split GROW centiles into multiple bins across a broader range of birthweights (Table 2). Relative to the 40-60th centile reference range, odds of stillbirth showed statistically significant increases beginning with the 35-39th centile and below and at the 91-95th centile and above. While statistically significant, the odds increase for most bins was modest. Xu et al.^16^ suggested an aOR of ≥ 2.0 as a heuristic to indicate a clinically meaningful odds increase; adapting this heuristic to our data, point estimates for increased odds of stillbirth in our study exceeded this threshold in the 5-9th centile bin and below for low birthweight and in the 96-100th centile bin for high birthweight. Plots depicting the predicted versus observed stillbirth rates from this model are provided in Supplementary Fig. S2 online. Results were similar to those described above, with a step function in predicted values that did not match changes in observed rates at the lowest and highest bin thresholds.

**Table 2:** Number of live births and late stillbirths, late stillbirth rate per 1000 births, and the unadjusted and adjusted odds ratios for GROW birthweight centile categories using 5-centile bins.

GROW centile bin	Live birth N	Stillbirth N	Stillbirth rate per 1000	OR (95% CI)	aOR (95% CI)
0–4	535,190	7478	13.78	**11.23 (10.62, 11.87)**	**9.65 (9.12, 10.21)**
5–9	379,588	1070	2.81	**2.27 (2.09, 2.45)**	**2.10 (1.94, 2.28)**
10–14	344,814	747	2.16	**1.74 (1.59, 1.90)**	**1.66 (1.52, 1.81)**
15–19	331,415	617	1.86	**1.50 (1.36, 1.64)**	**1.45 (1.32, 1.59)**
20–24	320,606	541	1.68	**1.36 (1.23, 1.50)**	**1.32 (1.20, 1.46)**
25–29	311,805	458	1.47	**1.18 (1.06, 1.31)**	**1.16 (1.04, 1.29)**
30–34	304,933	462	1.51	**1.22 (1.10, 1.35)**	**1.21 (1.09, 1.34)**
35–39	297,905	419	1.40	**1.13 (1.01, 1.26)**	**1.13 (1.01, 1.26)**
40–60	1,206,889	1502	1.24	Ref	Ref
61–65	280,790	348	1.24	1.00 (0.89, 1.12)	1.00 (0.89, 1.12)
66–70	281,155	360	1.28	1.03 (0.92, 1.15)	1.03 (0.92, 1.16)
71–75	281,558	367	1.30	1.05 (0.93, 1.17)	1.05 (0.93, 1.17)
76–80	286,806	376	1.31	1.05 (0.94, 1.18)	1.05 (0.94, 1.18)
81–85	294,213	383	1.30	1.05 (0.93, 1.17)	1.04 (0.93, 1.17)
86–90	309,722	418	1.35	1.08 (0.97, 1.21)	1.07 (0.96, 1.20)
91–95	350,782	572	1.63	**1.31 (1.19, 1.44)**	**1.27 (1.16, 1.40)**
96–100	613,879	2093	3.40	**2.74 (2.56, 2.93)**	**2.35 (2.20, 2.52)**

Numbers in bold indicate category is significantly different from the reference category (ref).

**Figure 4:**
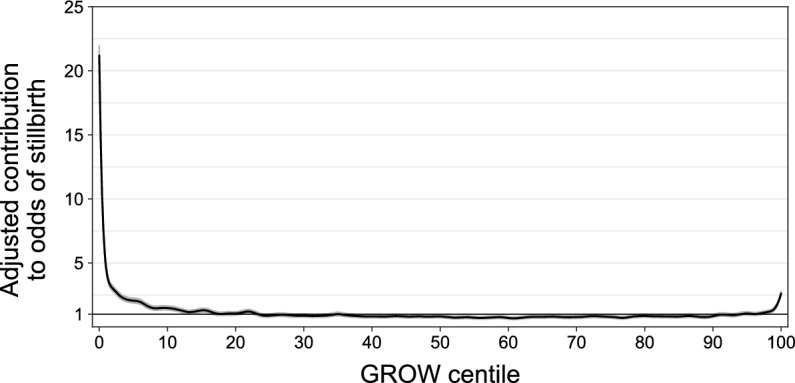
Results from multivariate GAM model. GAM smooth is shown on the odds (multiplicative) scale. Shaded areas reflect 95% confidence bands. Horizontal reference line shows adjusted odds contribution of 1.0, reflecting the dataset mean after covariate adjustment. Model is adjusted for mother’s race, mother’s Hispanic origin, mother’s age, mother’s prepregnancy BMI, mother’s educational attainment, mother’s nativity, father’s age, number of previous live births, infertility treatment, diabetes, hypertension, smoking, timing of prenatal care onset, infant sex, and WIC.

GAM results are depicted in Fig. 4. The smooth term for GROW centile is depicted on odds scale along with 95% confidence bands; a plot of the smooth term on the log odds scale is shown in Supplementary Fig. S3. 95% confidence bands for the adjusted contribution of GROW to stillbirth odds departed the baseline of 1.0 in the positive direction without re-crossing below 1.0 at the 16th centile for low birthweight and at the 98th centile for high birthweight. Note, however, that the baseline odds of 1.0 does not represent a researcher-defined reference, but rather the mean stillbirth odds in the dataset after adjustment for covariates, i.e., the model intercept. As such, these should be interpreted as the points beyond which the odds of stillbirth are statistically significantly higher than average. Although the contribution of birthweight centile to odds of stillbirth was significantly higher than the adjusted dataset average below the 16th centile, the increase was relatively small until the very low end of the birthweight centile range; the contribution to odds of stillbirth exceeded 2.0 above the dataset mean below the 5.7th centile at the low end and above the 99.7th centile at the high end. When decimating the continuous smooth function for GROW into centile-level values, the estimated adjusted contribution of GROW to odds of stillbirth in the 0th centile bin was 21.19-fold higher than the the adjusted mean (CI 20.43–21.98) and 2.61-fold (CI 2.47–2.77) in the 100th centile bin. Predicted stillbirth rates from the GAM, relative to observed stillbirth rates, are shown in Fig. 3b for the low end of the GROW continuum. Predicted rates show a good fit to the observed data across the range of birthweight centiles. This contrasts with the predicted/observed errors seen with the binned logistic regression using 10th/90th centile cutoffs (Fig. 3a). Plots showing the full GROW continuum are provided in Supplementary Fig. S1.

Observed stillbirth rates, predicted stillbirth rates from the GAM, and adjusted odds contribution estimates (with CIs) from the GAM by centile score bin for the full GROW continuum are shown in Table 3. Model fit metrics for the three models are shown in Table 4. As stillbirth is a rare event, all models showed very low sensitivity and high specificity. However, model discrimination measured by AUC increased modestly across the three increasingly complex models, with the GAM showing the best discrimination skill. PPV showed notable increases, indicating that the more complex models (multi-binned GLM and GAM) were more precise in identifying stillbirths than the baseline 3-bin GLM. Based on 95% CIs, AUC, sensitivity, and PPV were all statistically significantly improved in the GAM compared to the standard 3-bin GLM.

**Table 3:** Number of observations, observed stillbirth rate, and stillbirth model estimates from multivariate GAM using GROW centiles.

GROW centile	Number of observations	Stillbirth rate/1000 births		Estimated adjusted contribution to odds of stillbirth from GAM	
		Observed	Predicted	Estimate	95% CI
0	132,389	39.83	39.59	21.19	(20.44–21.97)
1	102,678	7.78	8.42	4.62	(4.32–4.93)
2	104,764	5.75	5.39	2.99	(2.77–3.22)
3	78,983	4.27	4.32	2.44	(2.23–2.67)
4	91,315	3.69	3.70	2.15	(1.96–2.35)
5	72,590	3.49	3.53	2.05	(1.86–2.26)
6	85,271	3.45	3.33	1.95	(1.77–2.16)
7	67,952	2.75	2.80	1.65	(1.48–1.84)
8	81,880	2.39	2.48	1.46	(1.31–1.63)
9	65,689	2.66	2.59	1.48	(1.32–1.66)
10	79,184	2.46	2.47	1.48	(1.32–1.65)
11	63,906	2.36	2.36	1.41	(1.25–1.59)
12	76,765	2.16	2.11	1.29	(1.14–1.45)
13	62,492	1.89	2.00	1.17	(1.03–1.32)
14	75,743	1.98	2.00	1.20	(1.06–1.36)
15	61,031	2.18	2.13	1.30	(1.14–1.47)
16	74,393	2.14	2.03	1.25	(1.10–1.41)
17	59,673	1.59	1.73	1.07	(0.93–1.22)
18	72,415	1.67	1.71	1.02	(0.89–1.16)
19	59,127	1.91	1.75	1.05	(0.92–1.21)
20	71,358	1.61	1.72	1.05	(0.92–1.20)
21	58,208	1.79	1.84	1.13	(0.99–1.30)
22	71,070	2.15	1.99	1.20	(1.06–1.37)
23	57,738	1.70	1.71	1.04	(0.91–1.20)
24	69,894	1.34	1.46	0.89	(0.77–1.03)
25	57,075	1.45	1.42	0.89	(0.76–1.03)
26	69,451	1.56	1.55	0.94	(0.81–1.08)
27	55,968	1.66	1.58	0.95	(0.82–1.10)
28	68,624	1.37	1.43	0.90	(0.78–1.04)
29	55,784	1.40	1.45	0.88	(0.76–1.02)
30	67,818	1.55	1.44	0.89	(0.77–1.03)
31	55,542	1.28	1.39	0.85	(0.73–0.99)
32	67,390	1.45	1.40	0.86	(0.74–1.00)
33	54,377	1.40	1.42	0.88	(0.75–1.02)
34	66,782	1.42	1.52	0.94	(0.81–1.08)
35	54,817	1.84	1.60	1.00	(0.87–1.16)
36	66,214	1.36	1.49	0.93	(0.81–1.08)
37	53,787	1.47	1.42	0.88	(0.76–1.02)
38	65,190	1.33	1.34	0.85	(0.73–0.98)
39	53,055	1.32	1.33	0.82	(0.70–0.96)
40	65,354	1.33	1.32	0.82	(0.70–0.95)
41	53,669	1.34	1.31	0.81	(0.69–0.95)
42	64,315	1.21	1.30	0.81	(0.70–0.95)
43	52,482	1.43	1.34	0.85	(0.73–0.99)
44	64,299	1.35	1.35	0.85	(0.73–0.98)
45	52,407	1.28	1.30	0.81	(0.69–0.95)
46	64,278	1.26	1.33	0.81	(0.70–0.95)
47	52,008	1.50	1.31	0.83	(0.71–0.98)
48	63,062	1.14	1.30	0.81	(0.69–0.94)
49	51,444	1.48	1.39	0.83	(0.71–0.97)
50	63,723	1.33	1.33	0.82	(0.71–0.96)
51	51,632	1.32	1.26	0.78	(0.66–0.91)
52	62,876	1.07	1.16	0.73	(0.62–0.86)
53	51,640	1.20	1.19	0.75	(0.63–0.88)
54	62,515	1.31	1.22	0.76	(0.65–0.90)
55	51,228	1.09	1.16	0.72	(0.61–0.85)
56	62,487	1.12	1.14	0.70	(0.60–0.83)
57	51,154	1.17	1.16	0.72	(0.61–0.86)
58	62,286	1.16	1.19	0.76	(0.64–0.89)
59	50,688	1.34	1.20	0.76	(0.64–0.90)
60	61,931	1.08	1.13	0.70	(0.59–0.83)
61	50,627	1.01	1.09	0.67	(0.57–0.80)
62	61,953	1.19	1.17	0.73	(0.62–0.86)
63	50,402	1.31	1.25	0.78	(0.67–0.92)
64	62,019	1.29	1.30	0.79	(0.68–0.93)
65	50,265	1.17	1.24	0.79	(0.67–0.93)
66	62,196	1.32	1.29	0.81	(0.70–0.95)
67	50,899	1.36	1.31	0.80	(0.68–0.94)
68	61,532	1.20	1.26	0.77	(0.66–0.90)
69	50,844	1.22	1.20	0.76	(0.65–0.90)
70	62,116	1.24	1.22	0.77	(0.66–0.91)
71	50,708	1.24	1.31	0.80	(0.68–0.94)
72	61,706	1.41	1.36	0.84	(0.72–0.99)
73	50,626	1.38	1.35	0.84	(0.72–0.99)
74	61,963	1.19	1.29	0.82	(0.70–0.95)
75	50,874	1.43	1.30	0.80	(0.68–0.94)
76	62,735	1.20	1.19	0.73	(0.62–0.86)
77	51,183	0.92	1.14	0.71	(0.60–0.84)
78	63,129	1.43	1.26	0.80	(0.68–0.94)
79	51,940	1.29	1.39	0.85	(0.72–0.99)
80	63,324	1.45	1.38	0.86	(0.74–1.01)
81	52,244	1.32	1.38	0.84	(0.72–0.98)
82	64,001	1.30	1.31	0.83	(0.71–0.97)
83	53,012	1.41	1.36	0.84	(0.71–0.98)
84	64,721	1.30	1.31	0.82	(0.70–0.95)
85	53,776	1.25	1.32	0.82	(0.70–0.95)
86	66,180	1.41	1.36	0.86	(0.74–1.00)
87	54,852	1.44	1.38	0.85	(0.73–0.99)
88	67,866	1.25	1.28	0.79	(0.68–0.92)
89	56,094	1.23	1.26	0.78	(0.66–0.91)
90	69,656	1.31	1.37	0.84	(0.73–0.98)
91	57,996	1.69	1.54	0.95	(0.82–1.10)
92	73,330	1.54	1.53	0.94	(0.82–1.09)
93	61,244	1.37	1.52	0.92	(0.80–1.06)
94	78,371	1.67	1.64	1.00	(0.88–1.14)
95	67,172	1.91	1.76	1.05	(0.92–1.20)
96	87,307	1.56	1.68	1.01	(0.89–1.14)
97	77,505	1.86	1.82	1.06	(0.94–1.20)
98	108,629	2.13	2.00	1.16	(1.05–1.30)
99	117,127	2.26	2.48	1.41	(1.28–1.55)
100	264,277	5.21	5.17	2.61	(2.47–2.77)

Adjusted odds contributions above and below 1 reflect odds above and below the dataset mean respectively, after adjustment for covariates. Different stillbirth rates and model estimates will be seen using other customized or uncustomised birthweight centile standards.

Predicted stillbirth rates reflect the mean predicted probability of stillbirth (× 1000) from the GAM for all infants within each GROW centile bin. Estimates from the GAM are adjusted for mother’s race, mother’s Hispanic origin, mother’s age, mother’s prepregnancy BMI, mother’s educational attainment, mother’s nativity, father’s age, number of previous live births, infertility treatment, diabetes, hypertension, smoking, timing of prenatal care onset, infant sex, and WIC.

**Table 4:** Model fit metrics. 95% CIs are in parentheses.

Model	AUC*	Sensitivity	Specificity	Positive predictive value
GLM 1 (3-bin)	0.80 (0.80–0.80)	0.01 (0.01–0.01)	> 0.99 (> − 0.99 to > 0.99)	0.42 (0.38–0.47)
GLM 2 (multi-bin)	0.81 (0.81–0.81)	0.01 (0.01–0.01)	> 0.99 (> − 0.99 to > 0.99)	0.46 (0.42–0.51)
GAM	0.82 (0.82–0.82)	0.02 (0.02–0.02)	> 0.99 (> − 0.99 to > 0.99)	0.53 (0.49–0.56)

*Area under the receiver operating characteristic curve.

## Discussion

This study characterized the relationship between fetal growth and risk of stillbirth. Consistent with previous research we found higher odds of stillbirth for infants at the low and high ends of the birthweight continuum, with the higher risk associated with SGA. However, although most studies use bins for high and low birthweight centiles as discrete categories, the distribution of stillbirth rates does not show corresponding discrete changes for any of the commonly used bin edges. For babies at the lower end of the birthweight continuum, small but statistically significant increases in odds of stillbirth begin in centiles much higher than the common 10th centile cutoff, and increases in odds show marked nonlinear accelerations toward the extreme ends of the continuum. As odds estimates in binned models reflect the average within the bins, categorical models underestimated stillbirth rates for the 0th and 100th centile values, but overestimated rates for other centile values within the SGA and LGA bins. This underestimation is particularly dramatic for the very smallest infants. As approximately 2% and 4% of infants in our cohort fell within the 0th and 100th centile bins, respectively, this underestimation in stillbirth rates applies to a relatively large number of births in our dataset.

In contrast to binned categorical models, stillbirth rate estimates from our GAM captured the nonlinear increases for both low and high birthweight babies; these rate estimates, which reflect the mean fitted stillbirth probability across observations within each centile bin, tracked closely with observed rates. Additionally, model fit metrics were significantly improved for the GAM compared to the 3-bin GLM. The estimated adjusted odds of stillbirth for babies in the 0th GROW centile from the GAM were approximately 21-fold higher than the adjusted dataset average odds, and the model-estimated stillbirth rate for this centile (39.6/1000 births) aligned closely with the observed rate (39.8/1000 births). This contrasts with the estimated approximate sixfold odds increase, relative to the reference bin, from the binned model using the common 10th centile cutoff, where the predicted stillbirth rate was 11.7/1000 births for the 0th centile. The standard binned models therefore greatly underestimated the risk facing the very smallest infants. Clinicians’ management of these pregnancies may be more conservative than is warranted when 4% will result in stillbirth.

Note that the risk increase seen with the largest infants was modest compared with the smallest infants and was restricted to only the very highest centiles; based on our GAM analysis, the contribution to an increase in odds of stillbirth only became statistically significantly higher than the adjusted mean at the 98th centile and above. Using standard ≥ 90th centile definition of LGA, a large number of babies would be categorized as LGA (15.7% of births) and therefore categorized as higher risk than would be based on our models, where only 7.1% of births fell in the 98^th^ centile and above, with an average stillbirth rate of 3.82/1000 in this group. Our results therefore suggest that clinical management of macrosomia with respect to stillbirth should focus on the very largest infants.

Although GAMs are able to provide flexible fits to continuous data, they do not inherently give specific cut-offs for categorizing pregnancies as high vs. average risk. Some prior work on stillbirth has suggested that infants should be considered at high risk of stillbirth or other poor perinatal outcomes beginning at birthweights much closer to the center of the birthweight continuum (e.g., 15th or 25th centile)^16,21^. For example, using neonatal death and Apgar score as proxies for fetal growth-associated perinatal risk, Xu et al.^16^ proposed a heuristic where birthweight centile bins with risk ratios ≥ 2.0 should be considered SGA; using this heuristic they suggested that the cutoff for SGA should be the 15th centile rather than the more commonly used 10th or 5th centiles. Although the details of their methods differed from ours, applying this heuristic to our findings would mean that infants at and below the 5-9th centile bin would be considered SGA, and therefore at high risk, based on the second GLM and below approximately the 5th centile based on the GAM. However, like the 10th/90th birthweight centile cutoffs for SGA and LGA, the ≥ 2.0 risk ratio cutoff is also heuristic and arbitrary. Recommending any specific threshold for defining high risk requires a balance between management that might include early delivery and its consequences, such as complications of prematurity, and the risk of fetal death.

One distinct advantage of using GAMs is that researchers are not inherently restricted to heuristic bin cut-offs for defining risk categories such as SGA; GAM estimates are based on smooth, continuous functions. Therefore, risk can be assessed individually based on the continuous estimates that GAMs provide. Practitioners can use the more precise risk estimates that GAMs provide for fetal growth in concert with other information, including gestational age and other factors, to assess risk on an individual basis. We therefore provide estimated risk coefficients for GROW centiles from the GAM in Table 3 for clinical reference.

Note that the specific stillbirth rates, model parameters, and model-derived risk estimates provided here used the GROW standard for birthweight centiles; if using any other birthweight standard, either customized or uncustomized, observed stillbirth rates, predicted stillbirth rates, and point estimates for stillbirth odds will differ. Nonetheless, whenever using hard bin cut-offs, the problem of poor stillbirth risk estimation at the extremes of the centile distribution and at the SGA and LGA bin edges that we have demonstrated here using GROW would be present regardless of the birthweight standard used, whether customized or uncustomized. This follows from the binning procedure itself. Risk estimation using GAMs could be applied with any set of birthweight charts to derive benefits in risk estimation similar to those we have demonstrated here.

One of the findings of this study was the non-uniform distribution of GROW values described above, where a bimodal distribution showed peaks at each end of the centile spectrum. 2% and 4% of births fell into the 0th and 100th GROW centile score bins, respectively. This bimodal distribution is to be expected as the GROW centile distribution was derived from uncomplicated pregnancies in healthy mothers and represent the optimal fetal growth. The ‘optimal weight’ is the weight the baby is predicted to achieve in the absence of pathological factors, such as smoking and diabetes. Maternal smoking is known to lower birthweight by 150 g and thus babies of mothers who smoke would be pushed into lower centiles than the expected optimal weight. Similarly maternal diabetes would result in increased fetal weight gain, thus resulting in higher centiles than the predicted optimal weight. Optimal weight is calculated using a fetal rather than a neonatal weight standard. Babies born preterm have lower birthweights than the weight of fetuses in utero at the same gestation that go on to deliver at term. Our study included babies born preterm which would have increased the number of subjects in the lower centiles. Furthermore, many stillbirth studies exclude congenital anomalies, many of which will have low birthweight. As the CDC does not provide information about congenital anomalies for stillbirths, we included all stillbirths except multiple births.

Limitations for this study have been described in detail previously^3^. Summarizing, there is some delay between fetal death and diagnosis/delivery. The CDC calculates gestation at time of birth, which will overestimate the length of gestation. This would bias centiles towards the low end^5^. To help account for potential delays, the GROW algorithm subtracts two days from the gestational age at birth in cases of fetal death, but this may be insufficient. Also, some data contained in the CDC files is given by maternal self-report, such as maternal smoking. Therefore there could be some errors or omissions in the data. However, these data were collected prior to the outcome and therefore are less likely to produce systematic bias. Some of these self-reported data were used in calculating GROW centile scores (maternal prepregnancy height and weight, parity, etc.) and were used as covariates in the statistical model. However, restricting our analyses to cases where CDC flags indicate high reporting quality mitigates this concern.

The approach we have taken to examine the relationship between fetal growth and stillbirth can be used to investigate other perinatal outcomes. The relationship between fetal growth and other perinatal outcomes could be different from that described here.

In conclusion, the relationship between fetal growth, measured by birthweight, and risk for stillbirth is continuous and non-linear. As a result, although they capture the general increased risk of stillbirth for SGA and LGA infants, standard risk models which define SGA and LGA with birthweight centile bins do so with poor granularity. This results in over-estimated stillbirth rates for most centiles within these high-risk bins and underestimated stillbirth rates for the extreme centile bins. In contrast, GAMs provided excellent estimates of stillbirth rates across the full birthweight spectrum. Our results suggest the stillbirth risk that the very smallest infants face is greater than much prior research has estimated, such that greater clinical attention should be paid to these infants. The risk charts we provide in Table 3 can be used by clinicians to assess risk at a more individualized level.

### Supplementary Information

Supplementary Information 1.

Supplementary Information 2.

## Data Availability

All data are available through the United States Centers for Disease Control and Prevention National Center for Health Statistics Vital Statistics Online Data Portal at https://www.cdc.gov/nchs/data_access/vitalstatsonline.htm.
