# Supplementary material for: Improved estimation of the relationship between fetal growth and late stillbirth in the United States, 2014–15
Source: Sci Rep. 2024 Mar 12;14:6002. doi: 10.1038/s41598-024-56572-7 (PMC10933328; doi:10.1038/s41598-024-56572-7)
Supplement: Supplementary file 1 — Supplementary Information 1. [file 41598_2024_56572_MOESM1_ESM.docx]

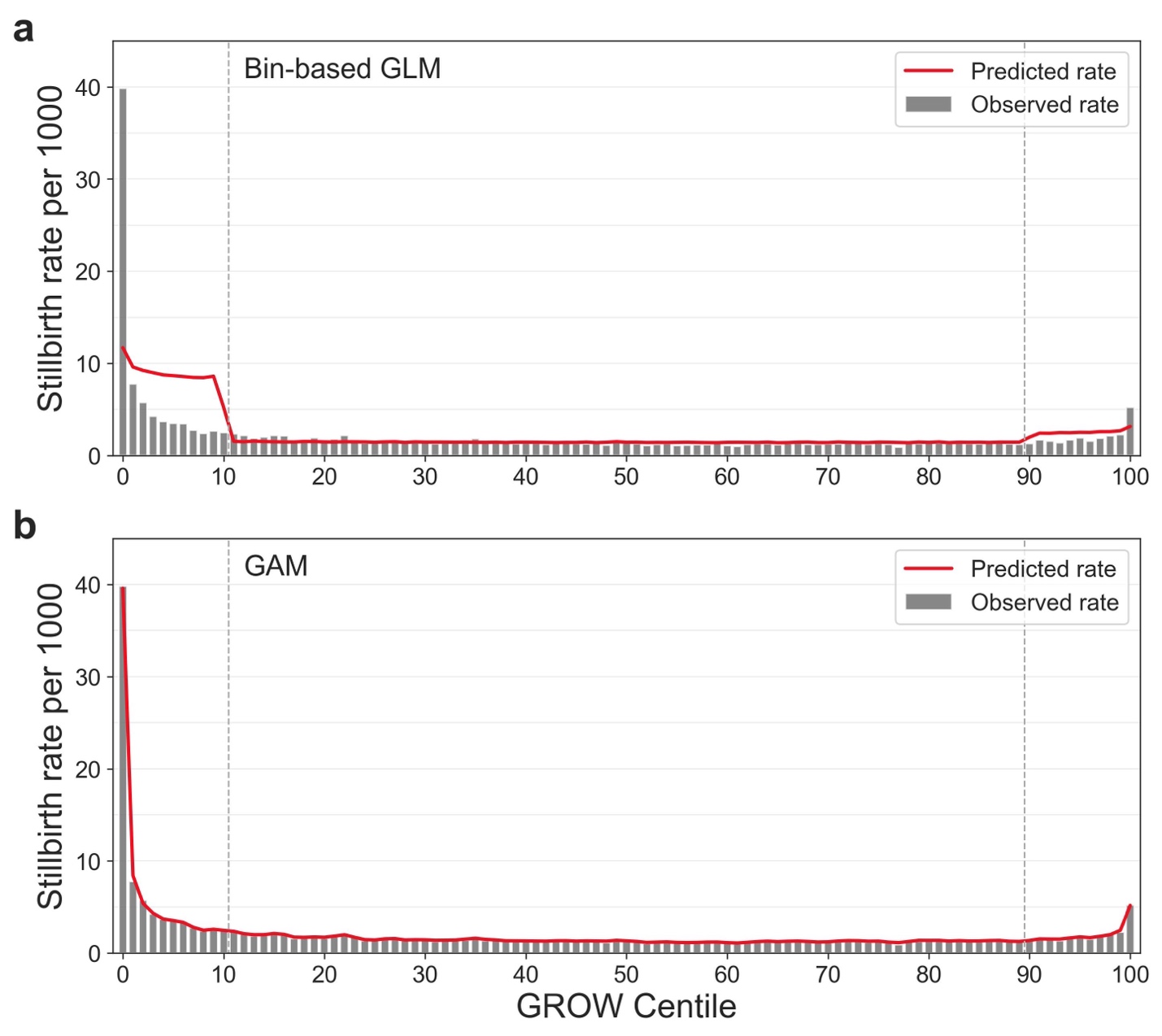


**Supplementary Figure S1. Observed and mean predicted stillbirth rates as a function of GROW centile.** (a) Observed rates (grey bars) and mean predicted rates (red line) derived from the bin-based logistic GLM using the 10^th^ and 90^th^ centile cutoffs to define SGA and LGA bins, respectively. (b) Observed rates (grey bars) and mean predicted rates (red line) derived from GAM. Vertical dashed lines depict the 10^th^ and 90^th^ centile bin cutoffs for SGA and LGA, respectively, used in the logistic GLM. Results are adjusted for mother’s race, mother’s Hispanic origin, mother’s age, mother’s prepregnancy BMI, mother’s educational attainment, mother’s nativity, father’s age, number of previous live births, infertility treatment, diabetes, hypertension, smoking, timing of prenatal care onset, infant sex, and WIC.


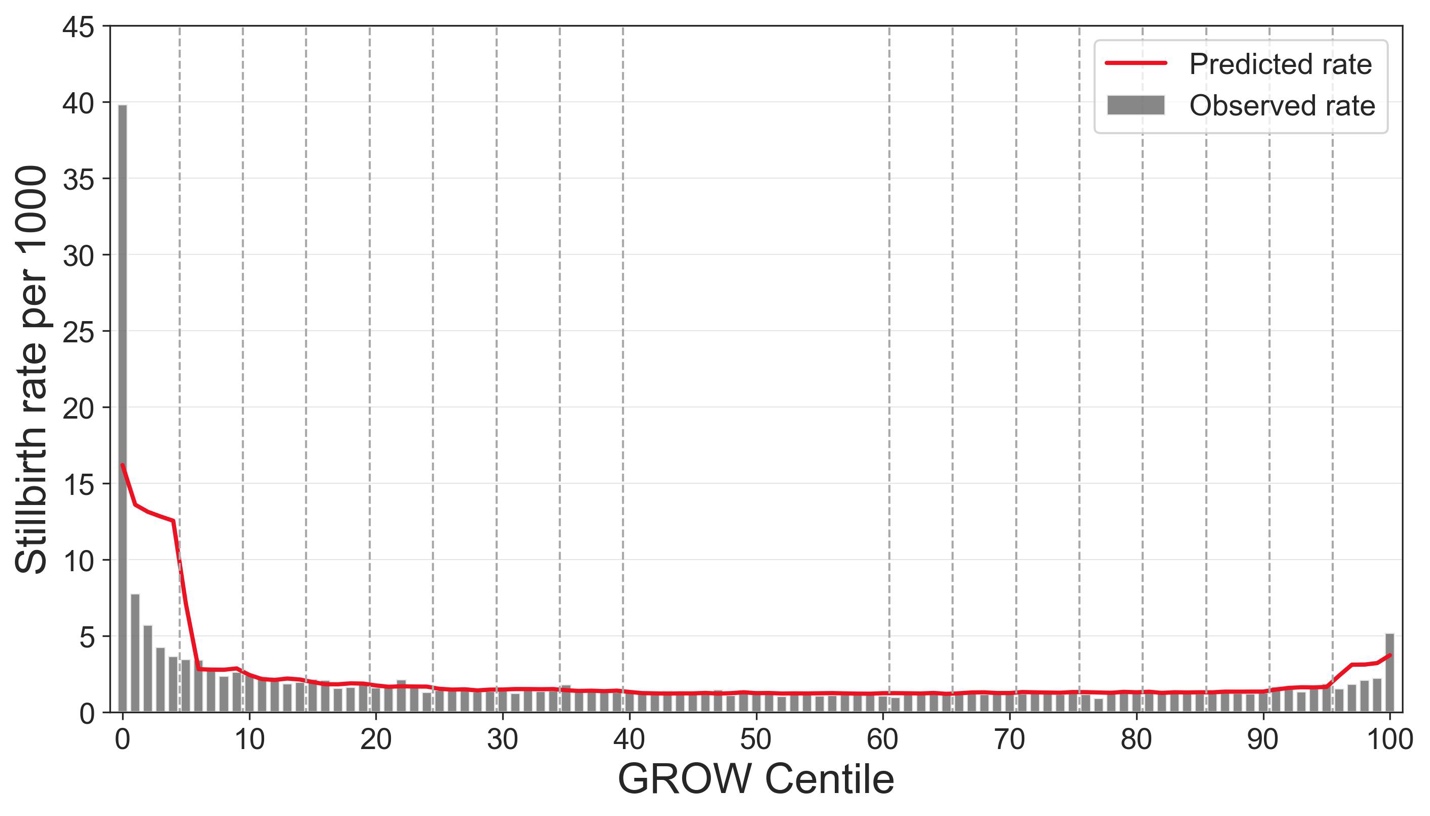


**Supplementary Figure S2. Observed and mean predicted stillbirth rates as a function of birthweight (GROW) centile for the second (5-centile bin) logistic GLM.** Observed rates are depicted by grey bars and predicted rates by the red line. Vertical dashed lines indicated the GROW bin edges used in the analysis.


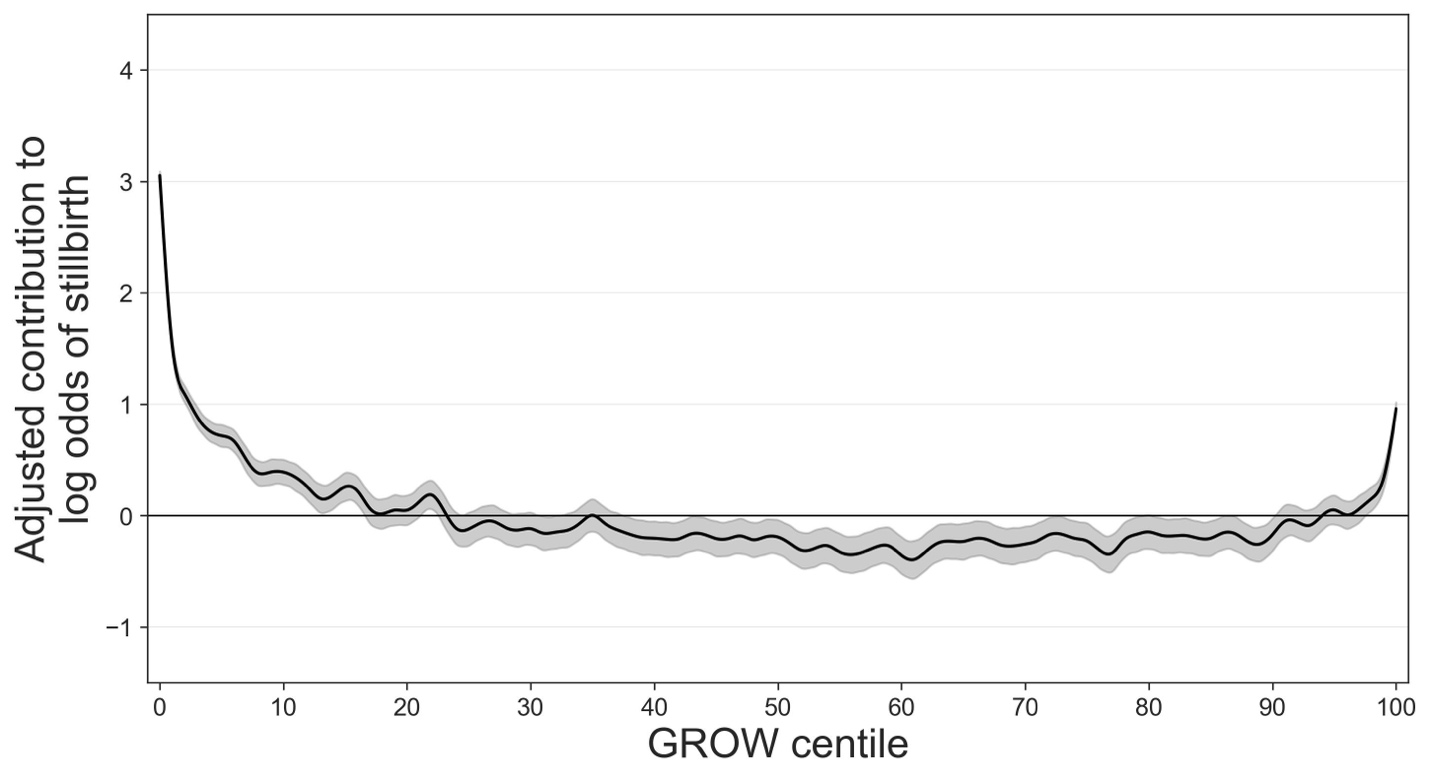


**Supplementary Figure S3. Results from multivariate GAM model.** GAM smooth is shown on the log-odds (additive) scale. Shaded areas reflect 95% confidence bands. Horizontal reference line shows baseline adjusted log odds contribution of 0.0, indicating the adjusted dataset mean. Results are adjusted for mother's race, mother's Hispanic origin, mother's age, mother’s prepregnancy BMI, mother's educational attainment, mother's nativity, father's age, number of previous live births, infertility treatment, diabetes, hypertension, smoking, timing of prenatal care onset, infant sex, and WIC.
